# A public health risk assessment for yellow fever vaccination: a model exemplified by an outbreak in the state of São Paulo, Brazil

**DOI:** 10.1590/0074-02760140345

**Published:** 2015-04

**Authors:** Ana Freitas Ribeiro, Ciléa Tengan, Helena Keico Sato, Roberta Spinola, Melissa Mascheretti, Ana Cecilia Costa França, Marcio Port-Carvalho, Mariza Pereira, Renato Pereira de Souza, Marcos Amaku, Marcelo Nascimento Burattini, Francisco Antonio Bezerra Coutinho, Luis Fernandez Lopez, Eduardo Massad

**Affiliations:** 1 Centro de Vigilância Epidemiológica, São Paulo, SP, Brasil; 2 Instituto Florestal, São Paulo, SP, Brasil; 3 Superintendência de Controle de Endemias, São Paulo, SP, Brasil; 4 Instituto Adolfo Lutz, São Paulo, SP, Brasil; 5 Faculdade de Medicina Veterinária e Zootecnia; 6 Faculdade de Medicina, Universidade de São Paulo, São Paulo, SP, Brasil; 7 Hospital São Paulo, Escola Paulista de Medicina, São Paulo, SP, Brasil; 8 Center for Internet Augmented Research and Assessment, Florida International University, Miami, FL, USA; 9 London School of Hygiene and Tropical Medicine, London, UK

**Keywords:** yellow fever, vaccination, side-effects, mathematical models, optimisation, risk estimation

## Abstract

We propose a method to analyse the 2009 outbreak in the region of Botucatu in the state of São Paulo (SP), Brazil, when 28 yellow fever (YF) cases were confirmed, including 11 deaths. At the time of the outbreak, the Secretary of Health of the State of São Paulo vaccinated one million people, causing the death of five individuals, an unprecedented number of YF vaccine-induced fatalities. We apply a mathematical model described previously to optimise the proportion of people who should be vaccinated to minimise the total number of deaths. The model was used to calculate the optimum proportion that should be vaccinated in the remaining, vaccine-free regions of SP, considering the risk of vaccine-induced fatalities and the risk of YF outbreaks in these regions.

Yellow fever (YF) is an haemorrhagic fever caused by the prototype member of the genus Flavivirus (a name which translates from Latin as "yellow virus") family Flaviridae (Monath 2001 , Moreno et al. 2011 ). This family comprises approximately 70 viruses (Moreno et al. 2011), most of which are transmitted by arthropod insects (hence the name arthropod born viruses or arboviruses). Genomic sequence analysis suggests that the YF virus evolved from other mosquito-borne viruses 3,000 years ago (Zanotto et al. 1996). It is an infection endemic in the tropical forests of Africa and Central and South America (Barnett 2004). In these regions, YF is enzootic and it is maintained by circulating in non-human primates and is transmitted by diurnally active mosquitoes (*Haemagogus *spp and *Sabethes *spp in the Americas and *Aedes *spp in Africa). During occupational or recreational activities in forest areas, susceptible individuals are bitten by mosquitoes carrying the YF virus and develop the clinical disease with varying degrees of severity. These individuals may eventually return infected to their home cities, which is of particular concern in urban regions infested by *Aedes aegypti* (YF vector of the urban cycle of the disease), the same transmitter of dengue fever and the mosquito responsible for huge YF outbreaks in urban centres in the past.

With 738 cases and 478 deaths, the last outbreak of urban YF in Brazil happened in Rio de Janeiro in 1928-1929 and in 1942, Brazil was declared free of YF by the Pan American Health Organization (Monath 1991). In 1958, the 20 year national campaign for the eradication of *Ae. aegypti* was successful and the mosquito disappeared from Brazilian cities. In 1967, the mosquito re-infested the states of Pará and Maranhão, but this re-infestation was short-lived and the mosquito was again eradicated in 1973. Unfortunately, in 1976 *Ae. aegypti* invaded Brazil again through the port of Salvador, state of Bahia, and spread throughout the country, infesting more than 80% of the Brazilian cities since then. Urban YF has never resurged and the diseased remained enzootic, causing occasional sporadic human cases (Costa et al. 2011, Moreno & Barata 2011).

Recently, however, YF re-emerged in the Central and Southeast Regions of Brazil, causing 51 cases and 21 deaths from 2008-2009 (Fortaleza et al. 2009). As a consequence, public health authorities intensified the YF vaccination programme. In the state of São Paulo (SP), approximately eight million doses of YF vaccine were administered between 1999-2008. This amount corresponds to approximately 19% of the entire state population (cve.saude.sp.gov.br/cgi/deftohtm.exe?cvetab/pnisp/pnidos.def).

In this paper, we propose a method to analyse the 2009 outbreak in the region of Botucatu, SP, when 28 YF cases were confirmed, including 11 deaths (Mascheretti et al. 2013). At the time of the outbreak, the Secretary of Health of the State of São Paulo vaccinated one million people, causing the death of five individuals, an unprecedented number of YF vaccine-induced fatalities. We apply a mathematical model described previously to optimise the proportion of people who should be vaccinated to minimise the total number of deaths (Massad et al. 2005 ). The model was used to calculate the optimum proportion that should be vaccinated in the remaining, vaccine-free regions of SP, considering the risk of vaccine-induced fatalities and the risk of YF outbreak in these regions. This work should be viewed as a methodological proposal rather than a definitive public health intervention policy.

## MATERIALS AND METHODS

If *p* is the proportion of the population that is pre-emptively vaccinated in campaigns before outbreaks, we can express the expected total number of deaths or serious adverse events, *D*(*p*), due to vaccination and potential YF outbreaks as (Bauch et al. 2003, Massad et al. 2005):

*D*(*p*) = *N* *_h _*{*pd* *_v_* *+ r*(1*- p*)[*π* *_yf _*(*p*)*d* *_yf_* *+ π* *_v_*(*p*)*d* ^*^ *_v _*(*p*)]}

where *N* *_h_* is the size of the human population, *d* *_v_* is the probability of developing serious adverse events (including deaths) after being pre-emptively vaccinated, *r* is the risk of an outbreak, *d* *_yf _*is the probability of dying of YF, p*_yf _*(*p*) is the probability of getting the infection if not vaccinated, *π* *_v_*(*p*) is the probability of receiving the vaccine during the outbreak and *d* ^*^ *_v _*(*p*) is the probability of developing serious adverse events (including death) from the vaccine received during the outbreak. The quantity *d* *_v _*, the risk of vaccine-induced fatality rate, was assumed to be equal to 5.0 x 10^-6^ doses^-1^, which was the value observed in 2009 in the region of Botucatu (Mascheretti et al. 2013). The risk of outbreak (*r*) is described below. The quantities *d* *_yf _*(*p*), *π* *_yf _*(*p*), *π* *_v_*(*p*) and *d* ^*^ *_v _*(*p*) were calculated through a dynamic system described in The dynamical system, Supplementary data. Therefore, we are considering the possibility of vaccination before and during an eventual outbreak.

The term N*_h_* *pd* *_v_* in equation is the number of serious adverse events (including deaths) of those individuals pre-emptively vaccinated in campaigns before outbreaks. The second term in equation, *N* *_h_* *r*(1*- p*)[(*d* *_yf _*(*p*) *-* *d* ^*^ *_v _*(*p*))*π* *_yf _*(*p*) + *π* *_v_*(*p*)*d* ^*^ *_v _*(*p*)] is the number of serious adverse events (including deaths) after an outbreak, due to death by YF infection and of serious adverse events (including deaths) due to vaccination during the outbreak.

We then minimise *D*(*p*) on the unit interval (0 ≤ *p *≤ 1) to determine the group optimum, *p* *_gr_*,which is the cover- age level that would have to be imposed to minimise the total expected number of serious adverse events (including deaths).

**Fig. 1:**
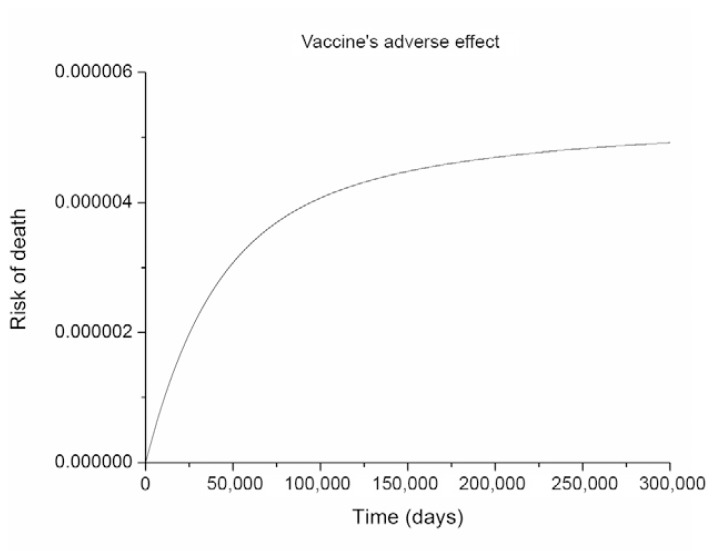
simulation of model (The dynamical system, Supplementary data) showing that the parameters chosen reproduce, in the equilibrium, the observed risk of vaccine-induced death after the Botucatu outbreak. The time scale is not relevant because the intent is just to demonstrate that the vaccination rate applied determined a lethality at equilibrium compatible with the one observed.

**Fig. 2:**
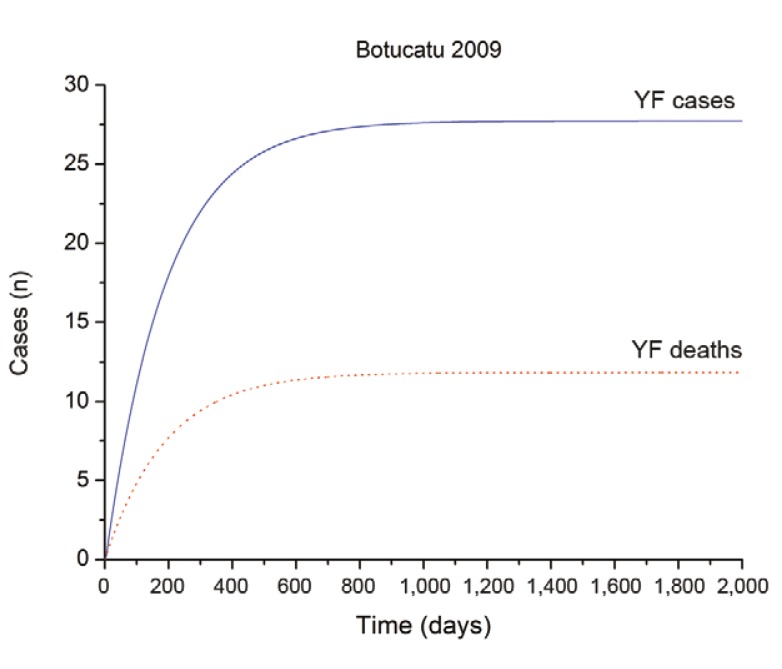
simulation of model (The dynamical system, Supplementary data) showing that the parameters chosen reproduce, in the equilibrium, the observed number of YF cases and deaths during the Botucatu outbreak.

**Fig. 3:**
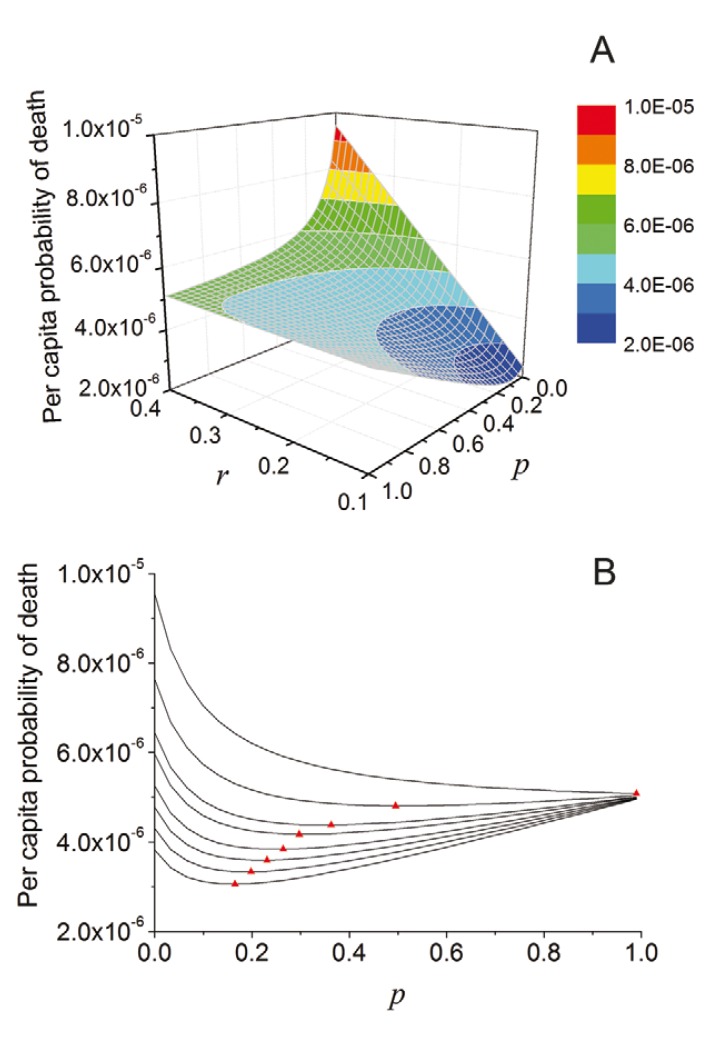
results of the simulation of D(p) / Nh from equation (main text), representing the per capita probability of death either by the disease or the vaccine. A: the three-dimensional figure showing D(p) / Nh as a function of proportion of preemptive vaccination, p, and the risk of outbreak, r; B: a profile of A, with only the risk of outbreaks, as in Supplementary Table II. The red triangles correspond to the minimum values of each curve.

The results of the equation simulation (Table I) are presented in Figs 1-3, considering the risk of vaccine-induced fatality rate of 5.0 x 10^-6^ dose^-1^ observed in 2009 in the region of Botucatu (Mascheretti et al. 2013) for various estimated risks of outbreaks (see below).

**TABLE I:** Parameters used in the simulations of modela

Parameter	Biological meaning	Value
*a*	Mosquitoes’ biting rate	0.78 day^-1^
*b*	Probability of transmission from infected mosquitoesto susceptible humans	1.0
γ^*h*^	Inverse of the intrinsic incubation period in humans	0.14 day^-1^
µ^*h*^	Natural mortality rate of humans	3.91 x 10^-5 ^day^-1^
a^*h*^	Yellow fever (YF)-induced mortality rate in humans	0.1 day^-1^
n^*h*^	YF vaccination rate	10^-5 ^day^-1^
µ^*v*^	Vaccine-induced mortality rate	2.1 x 10^-10 ^day^-1^
*c*	Probability of transmission from infected humans to susceptible mosquitoes	1.0
µ^*M*^	Natural mortality rate of mosquitoes	0.15 day^-1^
α^*M*^	YF-induced mortality rate in mosquitoes	0
τ	Extrinsic incubation period in mosquitoes	7 days
*p*	Proportion of preemptive vaccination	Variable

a: The dynamical system (Supplementary data).

We then applied the Delphi method to estimate the potential risk of outbreaks in several regions of SP (Dalkey & Helmer 1963, Massad et al. 1999, Ortega et al. 2003). The method was originally developed as a systematic, interactive forecasting method which relies on a panel of experts. The experts answer questionnaires in two or more rounds. After each round, a facilitator provides an anonymous summary of the experts' forecasts from the previous round as well as the reasons they provide for their judgments. Therefore, experts are encouraged to revise their earlier answers based on the replies of other members of their panel. During this process, the range of the answers will decrease and the group will converge towards the "correct" answer. Finally, the process is stopped after a pre-defined stop criterion (e.g., number of rounds, achievement of consensus and stability of results) and the mean or median scores of the final rounds determine the results.

During four meetings, the professionals of epidemiological surveillance, laboratory, vector control and the environment of the Centre of Epidemiological Surveillance, Adolpho Lutz Institute, Superintendence of Endemics Control and São Paulo Secretariat of the Environment discussed the risk of introduction of YF in new regions. We evaluated and assigned a score to define the risk in the epidemiological surveillance groups without recommendation of vaccination. The following variables were scored as 0, +1 or +2: presence of sylvatic vector, presence of *Ae. aegypti*, presence of non-human primates, dengue cases, river basin, climatic conditions, landscape, vegetation, fragmentation, proximity to the area of vaccine recommendation and rural population. The urbanisation density, the number of buildings per area of each region, was represented by the scores 0, -1 or -2. The eventual presence of non-explained deaths of non-human primates in any of the regions was not included in the risk analysis because, according to the current vaccination policy, if the non-human primate corpse is negative for the YF virus, then the risk is considered zero. If, in contrast, it is positive for YF, then the risk is considered 100% and the vaccination recommendation strategy is triggered. The results are shown in Table II.

**TABLE II:** Variables and values attributed by the Secretary of Health of the State of São Paulo staff through the Delphi method

Region	Variables												Total
	A	B	C	D	E	F	G	H	I	J	K	L	
Capital	1	1	2	1	0	1	1	2	2	0	0	-2	9
Santo André	1	1	1	1	0	1	1	2	2	0	0	-1	9
Mogi das Cruzes	1	1	2	1	0	1	1	2	2	0	0	-1	10
Franco da Rocha	1	1	2	1	0	1	1	2	2	0	0	0	11
Osasco	1	2	2	1	0	1	1	1	2	0	0	-1	10
Botucatu	2	2	2	2	2	2	2	2	2	2	2	0	22
Campinas	2	1	2	2	1	1	1	2	1	1	0	0	14
Piracicaba	2	2	1	2	2	2	1	1	0	2	0	0	15
Registro	2	1	2	1	1	2	1	2	2	2	2	0	18
Santos	2	2	2	2	0	2	1	2	2	0	0	-1	14
São João da Boa Vista	2	1	1	1	2	1	1	2	1	2	1	0	15
São José dos Campos	2	1	2	1	1	0	0	1	1	0	0	0	9
Taubaté	1	1	2	1	1	0	0	2	2	1	1	0	12
Sorocaba	2	2	2	1	2	1	1	2	1	2	2	0	18
Caraguatatuba	2	2	2	2	0	2	1	2	2	0	0	0	15

A: presence of sylvatic vectors; B: presence of Aedes aegypti; C: presence of non-human primates; D: cases of dengue; E: river basins; F: climatic conditions; G: landscape; H: vegetation; I: fragmentation; J: proximity to the area of vaccine recommendation; K: rural population; L: urbanization density.

Based on the Delphi scores, it was possible to estimate the relative risks of outbreaks for each region using the case of Botucatu as a reference. The risk assigned to Botucatu was equal to 40% or 0.4. This value was chosen because it is the minimum risk that resulted in 100% optimum vaccination coverage. In other words, the value of the risk assigned to Botucatu was such that no other risk value greater than that results in optimum coverage less than 100%. Therefore, it is the minimum risk that has 100% of coverage as the optimum level. According to Fig. 3B, we see that for 0% of vaccination, the total mortality in Botucatu (uppermost line) is approximately 11 (the actual observed value) and for 100% it was five (also the actual observed number of deaths due to the vaccine). All the other risks attributed to the regions were calculated as a linear proportion of the reference risk assigned to Botucatu. The results are shown in Supplementary Table I.

**Fig. 4:**
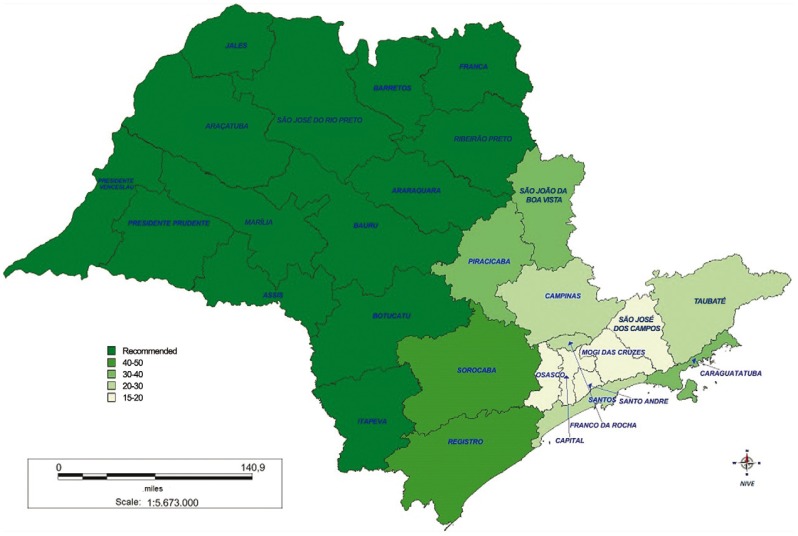
map of the state of São Paulo with the proposed optimal vaccination strategies for the unaffected areas. "Recommended" means indiscriminate vaccination of 100% of the area.

Fig. 4 shows the map of SP with the proposed optimal vaccination strategies for the unaffected areas. The calculated optimum proportion for vaccination against YF according to model (The dynamical system, Supplementary data) is shown in Supplementary Table II and Fig. 3A, B.

## DISCUSSION

Since its reintroduction in Brazilian regions in 1976, *Ae. aegypti* spread throughout the country, infesting more than 80% of urban centres (Costa et al. 2011).

The current circulation of YF virus in SP was associated with the high mobility of susceptible humans to enzootic YF areas. The virus eventually returned as viraemia to the *Ae. aegypti* infested home cities, which represents an enormous risk for urban YF re-emergence. Vaccination, therefore, is the main tool available to prevent the catastrophe represented by the re-emergence of urban YF. Even if we consider an unprecedented high rate of serious vaccine-induced adverse effects similar to the one observed in the Botucatu outbreak, vaccinating a high proportion of susceptible people in still unaffected areas should be indicated.

Presently, the surveillance policy against YF by the Brazilian Ministry of Health consists of maintaining high vaccination coverage of the so-called "recommendation" areas (dark green in Fig. 4) and triggering a mass vaccination action intended to cover entire areas where virus circulation is detected either in humans or non-human primates. This type of indiscriminate vaccination policy is far from optimum and therefore, an analysis such as the one proposed in this paper could help optimise the vaccination coverage necessary in each area.

Our results were based on the high level of vaccine-induced mortalities never observed before. If we consider the more accepted levels of vaccine-induced mortality proportions such as 2.5 deaths per one million doses as in Massad et al. (2005), the optimum vaccination doses required for minimising the total probability of death would be even higher than the levels we are proposing for the still unaffected areas. It is important to emphasise, however, that the aim of this paper was to propose a methodology to estimate the optimum vaccination strategies that would minimise the death both by the disease and by the vaccine.

In addition to the vaccine-induced mortality frequency, another critical parameter considered by our model is the expected risk of outbreak. We applied a relatively simple method for estimating those risks, but a more sophisticated analysis of the outbreak risk for each unaffected area would refine our results. Moreover, our model assumes various oversimplifications of the dynamics of YF such as the absence of an age component in transmission and several other sources of heterogeneities. The quality of the data used, collection system and its accuracy of association between vaccination and related deaths are weaknesses of our estimations. Our work, therefore, should be viewed as a methodological proposal rather than a definitive public health intervention policy.

If a vaccination strategy such as the one proposed in this paper is actually implemented in the regions where the optimum vaccination proportion is less than 100%, then the fraction of the population that should be vaccinated would be calculated by fractioning the risk regionally within the area.
